# Biocompatibility Assessment of a New Biodegradable Vascular Graft *viaIn Vitro* Co-culture Approaches and *In Vivo* Model

**DOI:** 10.1007/s10439-016-1601-y

**Published:** 2016-04-07

**Authors:** Marjan Enayati, Magdalena Eilenberg, Christian Grasl, Peter Riedl, Christoph Kaun, Barbara Messner, Ingrid Walter, Robert Liska, Heinrich Schima, Johann Wojta, Bruno K. Podesser, Helga Bergmeister

**Affiliations:** 1 Division of Biomedical Research, Medical University of Vienna, Vienna, Austria; 2 Ludwig Boltzmann Cluster for Cardiovascular Research, Vienna, Austria; 3 Centre of Medical Physics and Biomedical Engineering, Medical University of Vienna, Vienna, Austria; 4 Division of Internal Medicine II, Medical University of Vienna, Vienna, Austria; 5 Surgical Research Laboratories-Cardiac Surgery, Department of Surgery, Medical University of Vienna, Vienna, Austria; 6 Department of Pathobiology, Veterinary University, Vienna, Austria; 7 Institute of Applied Synthetic Chemistry, Technical University, Vienna, Austria

**Keywords:** Vascular graft, Biocompatibility, Biodegradable polyurethane, Fibroblast-macrophage co-culture

## Abstract

Following the implantation of biodegradable vascular grafts, macrophages and fibroblasts are the major two cell types recruited to the host-biomaterial interface. *In-vitro* biocompatibility assessment usually involves one cell type, predominantly macrophages. In this study, macrophage and fibroblast mono- and co-cultures, in paracrine and juxtacrine settings, were used to evaluate a new biodegradable thermoplastic polyurethane (TPU) vascular graft. Expanded-polytetrafluoroethylene (ePTFE) grafts served as controls. Pro/anti-inflammatory gene expression of macrophages and cytokines was assessed *in vitro* and compared to those of an *in vivo* rat model. Host cell infiltration and the type of proliferated cells was further studied *in vivo*. TPU grafts revealed superior support in cell attachment, infiltration and proliferation compared with ePTFE grafts. Expression of pro-inflammatory TNF-α/IL-1α cytokines was significantly higher in ePTFE, whereas the level of IL-10 was higher in TPU. Initial high expression of pro-inflammatory CCR7 macrophages was noted in TPU, however there was a clear transition from CCR7 to anti-inflammatory CD163 expression *in vitro* and *in vivo* only in TPU, confirming superior cell-biomaterial response. The co-culture models, especially the paracrine model, revealed higher fidelity to the immunomodulatory/biocompatibility behavior of degradable TPU grafts *in vivo*. This study established an exciting approach developing a co-culture model as a tool for biocompatibility evaluation of degradable biomaterials.

**Electronic supplementary material:**

The online version of this article (doi:10.1007/s10439-016-1601-y) contains supplementary material, which is available to authorized users.

## Introduction

Despite considerable research in the field of small diameter vascular graft (SDVG) replacement, there is still no ideal alternative to autografts up to now. Numerous problems are associated to synthetic SDVGs such as, thrombogenicity and intimal hyperplasia.^11^ Therefore, developing new generations of such conduits has become a major focus of scientists. One new approach is designing cell-free degradable vascular grafts, which promote remodeling *via* fast host cell infiltration. Furthermore, permanent presence of the foreign material in the host tissue will be avoided in these grafts.^22^ In our previous study a new biodegradable thermoplastic polyurethane polymer (TPU) with ultimate mechanical and functional behavior was synthesized and characterized.^4^ However, the inflammatory potential of the graft, which may evoke acute/chronic inflammatory responses at the site of implantation, still needs to be fully characterized.

Inflammation is the primary phase of wound healing and the presence of macrophages has a critical role for a positive healing outcome.^13^ Beside the presence of the macrophages, their activation state has equal importance on remodeling outcome. Activated pro-inflammatory macrophages, M1, secret inflammatory cytokines such as IL-1b, IL-1α, TNF-α and IL-6. While, activated anti-inflammatory macrophages, M2, express high level of IL-10 cytokine.^6^ An *in vitro* macrophage mono-culture model is one common method for evaluation of the host-biomaterial response. However, the extent of host-biomaterial response is defined by complex cell-based soluble signaling interactions between activated macrophages and other recruited cell types such as fibroblasts and endothelial cells at the graft-host interface.^23^ Among these cells, macrophages and fibroblasts are the major cells concurrently synchronizing and reacting to the foreign material *via* complex synergistic autocrine, paracrine (indirect cell contacts) and juxtacrine (direct cell contacts) signaling.^23^ Therefore, for a better cell-biomaterial interaction assessment, the effect of fibroblasts on macrophage polarization and modulation should be considered. However, there are only few studies on cooperative behavior of fibroblasts and macrophages *in vitro* in just juxtacrine co-culture model in the presence of biomaterials.^18^,^23^ It was shown that the magnitudes of the expression of cytokines in fibroblast-macrophage juxtacrine co-cultures were different from those in fibroblast/macrophage mono-cultures.^18^,^23^ Therefore, a better understanding of fibroblast and macrophage functional behavior *in vitro* in the presence of our TPU vascular graft in both juxtacrine and paracrine settings, could provide a better cell-biomaterial interaction assessment tool to study the immunomodulatory effect of the TPU biomaterial.

Fast host cell infiltration is essential for sufficient remodeling of a bioresorbable prosthesis. Following implantation, the composition of host-graft interface changes constantly. This leads to a continuous adoption of cells at the interface till the end of remodeling.^1^,^18^ The biomaterial should support host cell migration and proliferation and enable the presence of specific cellular phenotype necessary for remodeling.

This study seeks to evaluate the immunomodulatory behavior of degradable TPU grafts *via* fibroblast-macrophage paracrine (FM-Para-CC) and juxtacrine co-culture (FM-Jux-CC) models. TPU graft was compared with ePTFE graft, which is a clinically applied non-degradable synthetic vascular substitute. *In-vivo* immunomodulatory behavior of the grafts was compared with *in vitro* results. Furthermore, host cell infiltration into the TPU graft and the type of the proliferated cells were studied *in vivo*.

## Materials and Methods

As previously described, the prepolymer method was utilized to synthesize the new thermoplastic polyurethane polymer.^2^ TPUs are block copolymers containing hard- and soft-block sections with crystalline and amorphous domains. The material is based on classical poly(tetrahydrofuran) as soft-block and hexamethylene diisocyanate and bishydroxyethyl terephthalate as components for the hard-block in a molar ratio of 1:2:1. TPU grafts were fabricated *via* electrospinning and they were characterized morphologically and mechanically (fiber diameter; 1.39 ± 0.76 *µ*m, porosity; 74 ± 1%, pore size; 4.6 *µ*m, inner diameter; 1.6 mm, wall thickness; 78 ± 10 *µ*m).^4^ EPTFE grafts (inner diameter; 1.5 mm, wall thickness; 100 *μ*m, intermodal-distance; 5–25 *µ*m, Zeuss, Orangeburg, USA) were used as controls. Samples were sterilized with ethylene oxide before cell seeding and implantation. All experiments involving animals or animal tissues were conducted in compliance with European and national legislation and were permitted by the Animal Ethics Committee of the Medical University of Vienna and the Austrian Federal Ministry of Science and Research.

### Isolation of Primary Macrophages and Fibroblasts

Both macrophages and fibroblasts were isolated from four adult male Sprague–Dawley rats (350–400 g). Primary fibroblasts were isolated from rat skin biopsies as previously described.^23^ The skin samples were cut and placed on a petri dish with subcutaneous side down. Cells started to migrate out of the tissue after approximately 1 week incubation in UltraCULTURE™ medium (Lonza GmbH, Verviers, Belgium) including 10% fetal bovine serum (Millipore, Darmstadt, Germany) and 2% penicillin (Lonza GmbH, Basel, Switzerland). Subsequently, skin tissues were removed. The collected cells were passaged and passages 3–6 were used for the experiments. Isolated fibroblasts could be distinguished by their flat/spindle-shaped morphology and their branched cytoplasm surrounding an oval-shaped nucleus. Macrophages were isolated by a standard technique previously described.^23^ Briefly, peritoneal macrophages were obtained by lavage of the peritoneal cavity with 50 mL of cold phosphate-buffered saline (PBS, GIBCO^®^, Austria) and isolated by adherence on culture dishes for 1 h at 37 °C. Subsequently, cells were gently detached by rubber cell scraper and used for our studies.

### Mono- and Co-culture Models

The isolated cells were seeded on the luminal side of the grafts with the concentration of 2 × 10^5^ macrophages and 2 × 10^4^ fibroblasts per well (24 well plate) for the macrophage and fibroblast mono-cultures. Mixtures of macrophages and fibroblasts (2 × 10^5^ M + 2 × 10^4^ F) were used for the juxtacrine co-culture models. For paracrine co-culture, 0.4 *μ*m pore transwell^®^ polyester membrane cell culture inserts (Corning, USA) for 24 well plates were utilized. 2 × 10^5^ macrophages per well were seeded on the grafts and 2 × 10^4^ fibroblasts were seeded on each transwell insert. Culture media that were used for all the mono and co-cultures were similar to the media used for cell isolations. Cells were cultured on NUC™ Thermanox™ plastic coverslips (Thermo Scientist, NY, USA) as control groups.

### Cell Morphology Studies

Morphology and attachment of the macrophages and fibroblasts to the grafts were visualized *via* crystal violet (CV) staining and scanning electron microscopy (SEM, JEOL JSM-5400, JEOL Ltd., Japan) in mono- and co-cultured models after 7 and 21 days. Briefly, for CV staining, samples were fixed with paraformaldehyde (3.7%) and subsequently incubated with 0.05% crystal violet prior to microscopy. For SEM, samples were fixed in 2.5% glutaraldehyde. They were dried using hexamethyldisilazane (reagent grade ≥99%, Sigma Aldrich, Austria) and then sputter coated with gold prior to SEM.

### Viability and Proliferation Studies

The proliferation of the fibroblasts in mono and co-cultures was investigated using a XTT cell viability and proliferation kit (Biomol, Hamburg, Germany) after 7, 14 and 21 days. 208 *μ*L XTT was added, and the scaffolds including adherent cells were incubated for 20 h. Absorption of the reduced XTT was measured at 450 nm (reference wavelength 595 nm) on a Victor3 spectrophotometer (VICTOR3, Perkin Elmer, MA, USA). Grafts incubated with culture media without cells served as blank and data has been adjusted to the blank. All experiments were repeated independently 3 times (*n* = 3 per time-point per group).

### Cell Distribution Study

3D distribution of the cells and population of the positive cells on the grafts was investigated using confocal laser-scanning microscope (CLSM 700, Zeiss, Germany) after 21 days. Mouse-anti-rat-CD68 (pan macrophages) and mouse-anti-rat-CD163 (M2 anti-inflammatory macrophages) primary antibodies (both from AbD Serotec, Duesseldorf, Germany) were utilized. Briefly, after fixing the cells with paraformaldehyde (3.7%), non-specific sites were blocked with 1.5% normal goat serum (Dako, Glostrup, Denmark) and then the samples were stained with the primary antibodies. Subsequently, the samples were incubated with secondary antibodies (Goat anti-mouse igG Alexa Fluor 488, abcam, Bristol, UK,) with the dilution of 1:300 in PBS. Nuclei of the cells were counterstained with DAPI using Prolong^®^ Gold antifade embedding solution containing DAPI (Life Technologies, USA). For quantification of the positive cells, five random positions of each samples with the area of 2 mm × 2 mm and thickness of 100 *µ*m have been selected and scanned (*n* = 3 per group).

### Real-Time PCR

Real-time PCR was used to identify the differential expression of CD68, CCR7 and CD163 macrophage markers (7, 14 and 21 days) and the IL-1α, IL-10 and TNF-α cytokines (2, 24, 48 and 72 h,) in the presence of the TPU and ePTFE grafts. Total cellular RNA was isolated using the RNeasy Mini Kit (Qiagen, Valencia, CA) *via* QIAcube system (Qiagen, Valencia, CA). Reverse transcription was performed using GoScript™ Reverse Transcription System (Promega, Austria). Finally, the real-time PCR was performed with a Roche light cycler 480 (Basel, Switzerland). Primers were designed using the UniversalProbeLibrary Assay Design Center. Table 1 shows the specifications of primers. Data were analyzed using LightCycler Software (LightCycler Software Version 3.5, Roche, Basel, Switzerland). Cells cultured on plastic coverslips were considered as negative controls. Lipopolysaccharide (LPS, concentration 10 *μ*g/mL, Sigma, Austria) stimulated cells were our positive controls in cytokine release studies. The GAPDH housekeeping gene was used as a reference. Furthermore, the ratio of CCR7/CD163 gene expression was calculated as an indicator of M1/M2 response. Values more than 1.0 were representative of the predominance of M1 response whereas, a value of less than 1.0 was representative of a predominance of M2 response. All experiments were repeated independently 3 times (*n* = 3 per time-point per group).

**Table 1:** Specifications of the primers used.

Gene	Forward primer	Reverse primer	UPLprobe	Amplicon Size (bp)
GAPDH	tgggaagctggtcatcaac	gcatcaccccatttgatgtt	# 9	78
IL-1α	aaatactcagctctttgtgagtgc	tgtgatgagttttggtgtttcc	#7	85
TNF-α	tgaacttcggggtgatcg	gggcttgtcactcgagtttt	#63	122
IL-10	cagattccttactgcaggacttta	caaatgctccttgatttctgg	#13	128
CD68	acggacagcttacctttgga	aatgtccactgtgctgcttg	#21	118
CD163	atggggaaggcacaactg	tcagatccgctccgtctaa	#73	68
CCR7	tggctctcctggtcattttc	gccgatgtagtcgtctgtga	#29	65

### *In-Vivo* Implantation

TPU and ePTFE grafts (ID: 1.5 mm, length: 2 cm) were implanted into the infrarenal aorta of male Sprague–Dawley rats (300–400 g, *n* = 8 animals per time-point per group) using microsurgical techniques as previously described.^4^ No anti-coagulation nor anti-platelet drugs were administered. TPU and ePTFE grafts were harvested after 1 week and 1 month for PCR and histological assessments (*n* = 4 per time-point per group for each assessment).

#### Real-Time PCR

RNA isolation, CDNAs and PCR protocols were identical to that used in *in vitro* studies (“Real-Time PCR” section).

#### Histological Assessment

Following dehydration of formaldehyde-fixed samples, they were embedded in paraffin. Histological cross-sections (3 *µ*m) were processed for hematoxylin and eosin (H&E) staining. For characterization of proliferated cells and macrophages, sections were incubated with the monoclonal mouse-anti-Ki67 (clone MM1, Leica Biosystems, Nussloch, Germany), monoclonal mouse-anti-rat CD 68 (clone ED1, AbD Serotec, Oxford, UK) and monoclonal mouse-anti-rat CD163 (clone ED2, AbD Serotec, Oxford, UK) primary antibodies. Following washing steps, slides were incubated with the secondary antibody BrightVision Poly-HRP-anti-mouse (Immunologic, Duiven, The Netherlands). Images were captured using an Olympus BX microscope.

Double immunofluorescence staining was carried out to specifically identify the number of Ki67, desmin, α-SMA, PDGF, VEGF positive cells and to assess the percentage of desmin/α-SMA/PDGF/VEGF positive cells within the proliferated Ki67^+^ cells. To do so, following primary antibodies in combination with Ki67 have been used: monoclonal mouse-anti-smooth muscle actin (clone 1A4, DAKO, Glostrup, Denmark), monoclonal mouse-anti-desmin (clone D33, DAKO, Glostrup, Denmark), polyclonal rabbit anti-vascular endothelial growth factor A (Santa Cruz Biotechnology, Dallas, USA) and polyclonal rabbit anti-platelet derived growth factor (PDGFBB, Abcam, Cambridge, UK). Stainings were visualized using AlexaFluor goat-anti-mouse 568 (Molecular Probes) dilution 1:100 in PBS (for Ki67 positive cells), AlexaFluor goat-anti-mouse 488 (Molecular Probes) diluted 1:100 in PBS for α-SMA and desmin positive cells and AlexaFluor goat-anti-rabbit 488 (Molecular Probes) diluted 1:100 in PBS for PDGF and VEGF positive cells. Nuclei were stained by incubating in 4′,6-diamidino-2-phenylindole (DAPI, Sigma Aldrich, St. Louis, MO, USA). Immunofluorescent double-stained sections were evaluated *via* a confocal laser-scanning microscope (Zeiss, Germany, LSM 700).

#### Quantification and Analyzes of the Images

Three sections of midgraft region from each sample were randomly selected. The TissueQuest Analysis software (TissueGnostics GmbH, Austria) was used to count Ki67, CD68, CD163, desmin, α-SMA, PDGF and VEGF positive cells. The percentage of double positive cells were assessed by detecting the positive staining in the cytoplasma within a ring mask, which was grown from the nucleus to a defined distance.

### Statistical Analysis

All data were expressed as mean ± standard deviation (SD). Statistical analyzes were performed using GraphPad Prism 6 software (GraphPad Software Inc., CA, USA). Two-way analysis of variance (ANOVA) using Bonferroni *post hoc* tests were applied for multiple and pair-wise comparisons between the groups. Statistical significance was defined for a *p* value of < 0.05.

## Results

### Cell Attachment, Morphology, Viability and Proliferation

**Figure 1:**
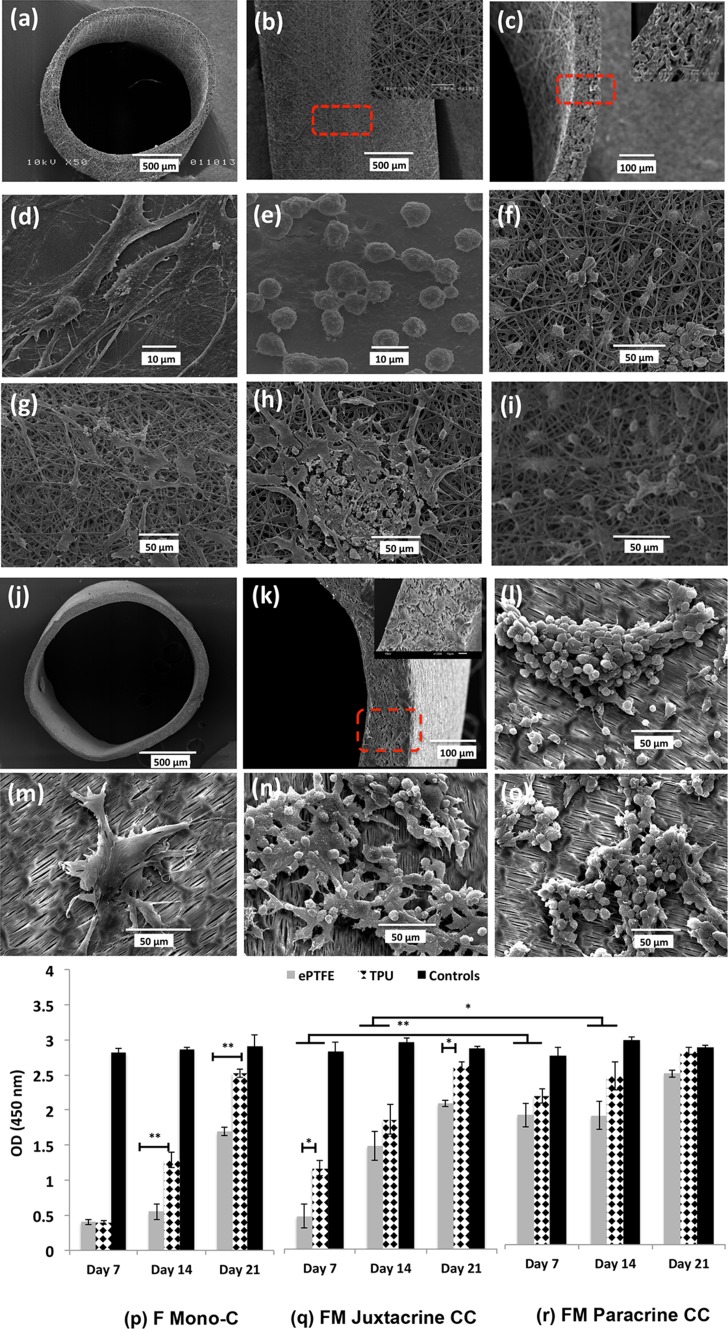
Morphological and cell viability/proliferation studies. SEM micrographs of TPU/ePTFE grafts and morphology of the isolated primary fibroblasts and macrophages seeded on coverslips as control and on TPU/ePTFE grafts in mono-cultures and co-culture models, after 21 days. (a) TPU graft, (b) adventitial surface of the TPU graft and close up of the surface, (c) cross-section and close up of the wall structure, (d) primary fibroblasts (e) primary macrophages cultivated on plastic coverslips, (f) fibroblast mono-culture, (g) macrophage mono-culture, (h) fibroblast-macrophage juxtacrine co-culture and (i) macrophages in the fibroblast-macrophage paracrine co-culture after 21 days in TPU grafts. (j) ePTFE graft, (k) cross-section and close up of the wall structure, (l) macrophage mono-culture, (m) fibroblast mono-culture, (n) fibroblast-macrophage juxtacrine co-culture and (o) macrophages in the fibroblast-macrophage paracrine co-culture after 21 days, in ePTFE grafts. (p-r) Proliferation of fibroblast cells at different time points (7, 14 and 21 days), on TPU and ePTFE grafts in (p) fibroblast mono-culture, (q) fibroblast-macrophage juxtacrine co-culture and (r) fibroblasts seeded on transwell membrane in fibroblast-macrophage paracrine co-culture models. Data represent mean ± S.D. (*n* = 3 per time-point per group, technical replicates: 3). *p < 0.05, **p < 0.001.

TPU electrospun grafts showed randomly oriented fibers (Figs. 1a–1c). Both fibroblasts and macrophages attached to the TPU grafts and no significant changes in morphology were apparent compared to the graft-free cultures (Figs. 1d–1i). The morphology of macrophages and fibroblasts did not change in the co-cultures, suggesting that these two cell types had no obvious effect on the structure of one-another. Morphology and cross-section of ePTFE grafts with the wall thickness of 100 *μ*m were shown in Figs. 1j–1k. Further crystal violet staining and SEM images showed that ePTFE grafts did not support the cell attachment as good as TPU grafts and cells were not homogenously distributed on the ePTFE grafts. Cell clusters as seen in ePTFE may be attributed to the more hydrophobic characteristics of this graft compared to TPU graft (Figs. 1l–1o, supplementary Fig. 1).^4^

The influence of macrophages on proliferation of fibroblasts was further investigated (Figs. 1p–1r). Proliferation of fibroblasts increased significantly from day 7 till day 21 in all cultures in both TPU (*p* < 0.001 in all cultures) and ePTFE grafts (*p* < 0.001 in F-C and FM-Jux-CC and *p* < 0.05 in FM-Para-CC). In general, co-cultures showed higher proliferation compared to F mono-cultures. Comparing juxtacrine with paracrine co-cultures, the proliferation of the fibroblasts in paracrine co-culture was significantly higher than in juxtacrine at earlier time points (*p* < 0.001; day 7 and *p* < 0.05; day 14) in both grafts. This suggests that paracrine model could better support the fibroblast proliferation. Comparing TPU with ePTFE grafts, proliferation of fibroblasts was higher in F mono-culture and FM co-culture models in TPU grafts.

### Cell Distribution Studies *In Vitro*

**Figure 2:**
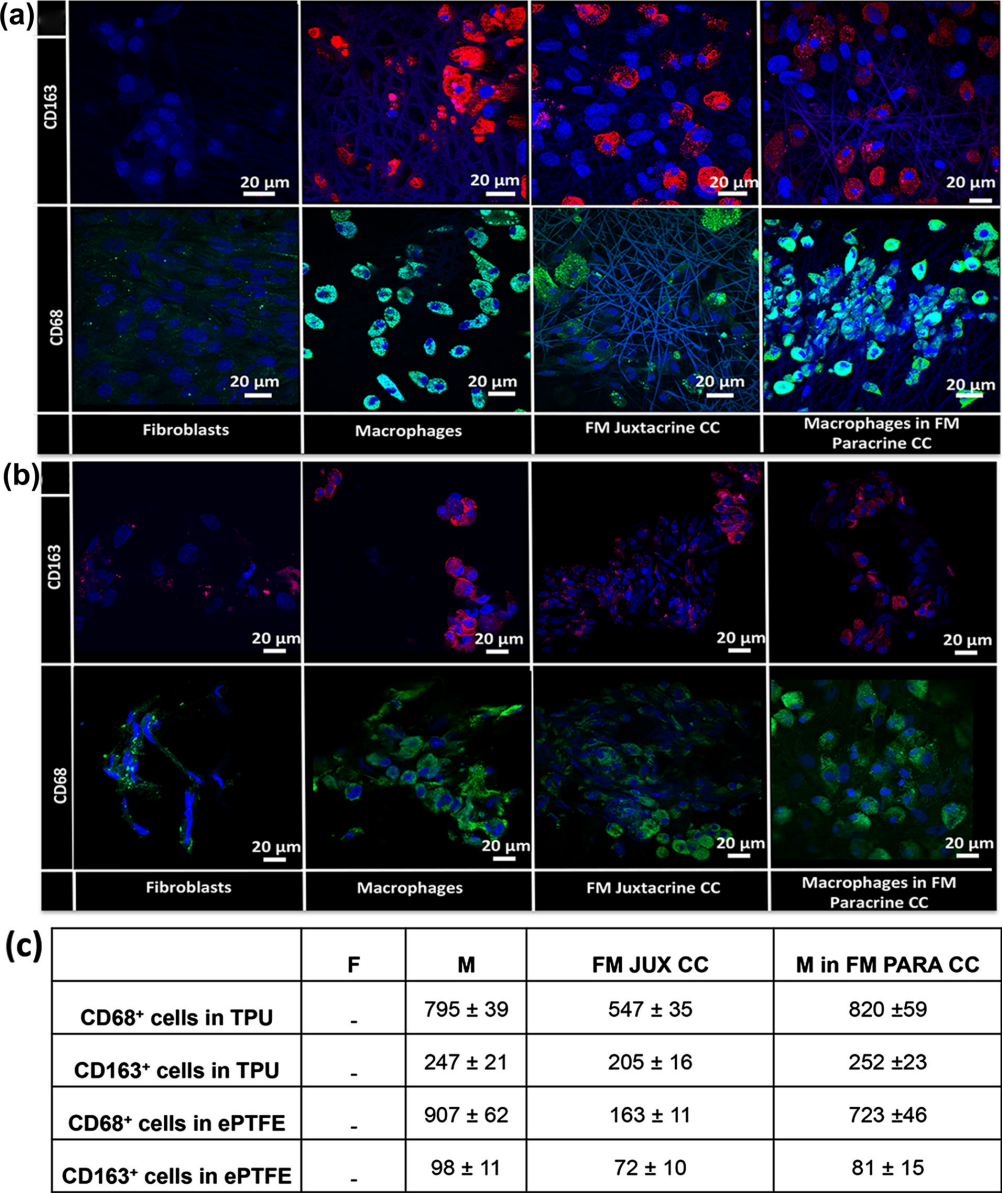
Cell distribution studies and quantification of the positive cells *via* confocal microscopy. CD68 (green) and CD163 (red) immunofluorescence staining of macrophage and fibroblast mono-cultures and fibroblast-macrophage juxtacrine (FM Juxtacrine CC) and paracrine co-cultures (FM Paracrine CC) in (a) TPU and (b) ePTFE grafts after 21 days. The nuclei of cells were counterstained with DAPI (blue). (c) Quantification of the CD68 and CD163 positive cells on TPU and ePTFE grafts after 21 days in macrophage (M) and fibroblast (F) mono-cultures and fibroblast-macrophage juxtacrine (FM JUX CC) and paracrine co-cultures (FM PAR CC). Data are presented as mean ± standard deviation (*n* = 3 per group).

Macrophages were stained with CD68 (pan macrophage) and CD163 (M2 anti-inflammatory) macrophage markers to visualize the distribution of macrophages and to distinguish the M2 anti-inflammatory macrophages in the mono and co-cultures (Figs. 2a, 2b). Fibroblasts were not positive for both markers. CD68 and CD163 positive macrophages were evenly distributed inside the TPU grafts. While in ePTFE grafts, cells were located mostly on the surface of the grafts and very few cells migrated into the grafts. Furthermore, there were less anti-inflammatory CD163 positive cells in ePTFE grafts compared with TPU grafts (Fig. 2c).

### Cytokine Expression

**Figure 3:**
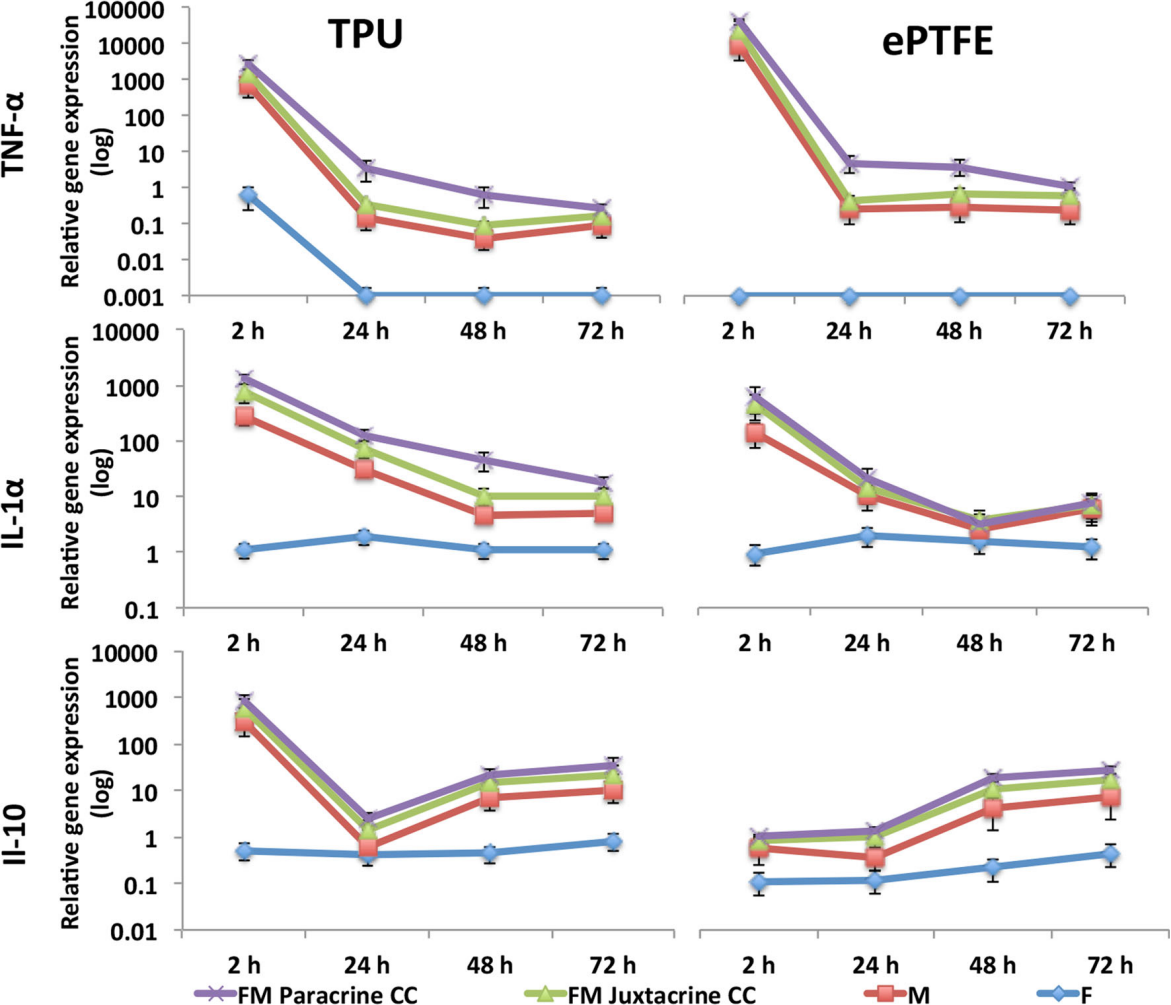
Early inflammatory gene expressions of TNF-α, IL-1α and IL-10 cytokines in the presence of TPU and ePTFE grafts in macrophage and fibroblast mono-cultures and macrophage-fibroblast juxtacrine (FM Juxtacrine CC) and paracrine co-culture (FM Paracrine CC) models, at different time points. The expression levels of all marker genes were normalized to the expression levels of GAPDH. Data are presented as mean ± standard deviation (*n* = 3 per time-point per group, technical replicates: 3). (For better representation of the data, logarithmic scales have been used).

The early inflammatory responses of TPU and ePTFE grafts to fibroblasts and macrophages were investigated after 2, 24, 48 and 72 h (Fig. 3). LPS stimulated macrophages and fibroblasts (positive controls) revealed the maximum expression of cytokines in macrophage mono-culture and fibroblast-macrophage co-cultures (data not shown). In general, pro-inflammatory response to the TPU and ePTFE grafts were apparent after 2 h. However, high expression levels of cytokines after only 2 h may be affiliated to the ongoing stress of the seeding process, even though cells had attached to the surface. Expression of pro-inflammatory TNF-α was significantly higher than anti-inflammatory IL-10 in both grafts (*p* < 0.001) after 2 h. However, expression of both TNF-α and IL-1α was significantly down-regulated after 48 and 72 h in both materials (*p* < 0.001). At final time-point (72 h), the level of anti-inflammatory IL-10 was significantly higher than pro-inflammatory IL-1α (*p* < 0.05) only in TPU grafts.

#### Comparison of FM Co-cultures vs. Mono-cultures

Expression of pro- and anti-inflammatory cytokines was not prominent in the fibroblast mono-cultures. However, the presence of fibroblasts in co-cultures had evident influence on expression of these cytokines. The expression of TNF-α and IL-1α pro-inflammatory cytokines was higher in FM co-culture models compared with M mono-cultures and this was more significant in paracrine co-cultures (TPU; M vs. FM-Para, TNF-α and IL-1α: 2, 24, 48, 72 h: *p* < 0.001), (ePTFE; M vs. FM-Para, TNF-α: 2, 24, 48, 72 h: *p* < 0.001, IL-1α: 2 h: *p* < 0.001, IL-1α: 24, 48, 72 h: *p* < 0.05).

#### Comparison of FM Paracrine vs. Juxtacrine Co-cultures

The trend of cytokines expressions was similar in both paracrine and juxtacrine co-cultures. However, in the paracrine model the expressions of TNF-α and IL-1α pro-inflammatory cytokines were higher at all time points compared to juxtacrine model (TPU; FM-Jux vs. FM-Para, TNF-α, IL-1α: 2, 24, 48 h: *p* < 0.001), (ePTFE; FM-Jux vs. FM-Para, TNF-α: 2, 24, 48 h: *p* < 0.001, IL-1α: 2, 24 h: *p* < 0.05). Expression of anti-inflammatory IL-10 did not show significant differences between paracrine and juxtacrine co-cultures.

#### Comparison of TPU vs. ePTFE Grafts

Both grafts showed high expression of pro-inflammatory cytokines at the beginning. These expressions were then followed by significant down-regulation of pro-inflammatory and up-regulation of anti-inflammatory IL-10 cytokines (after 24 h). The level of anti-inflammatory IL-10 was significantly higher in TPU grafts compared with ePTFE grafts in both M mono-culture (after 2, 48, 72 h) and FM co-cultures (after 2, 24, 72 h). Furthermore, level of pro-inflammatory IL-1α and TNF-α were significantly higher in ePTFE grafts compared with TPU in co-culture models, (IL-1α:2 h: *p* < 0.001, 72 h: *p* < 0.05 and TNF-α: 2, 48, 72 h: *p* < 0.001).

### Macrophages Gene Expression Studies

**Figure 4:**
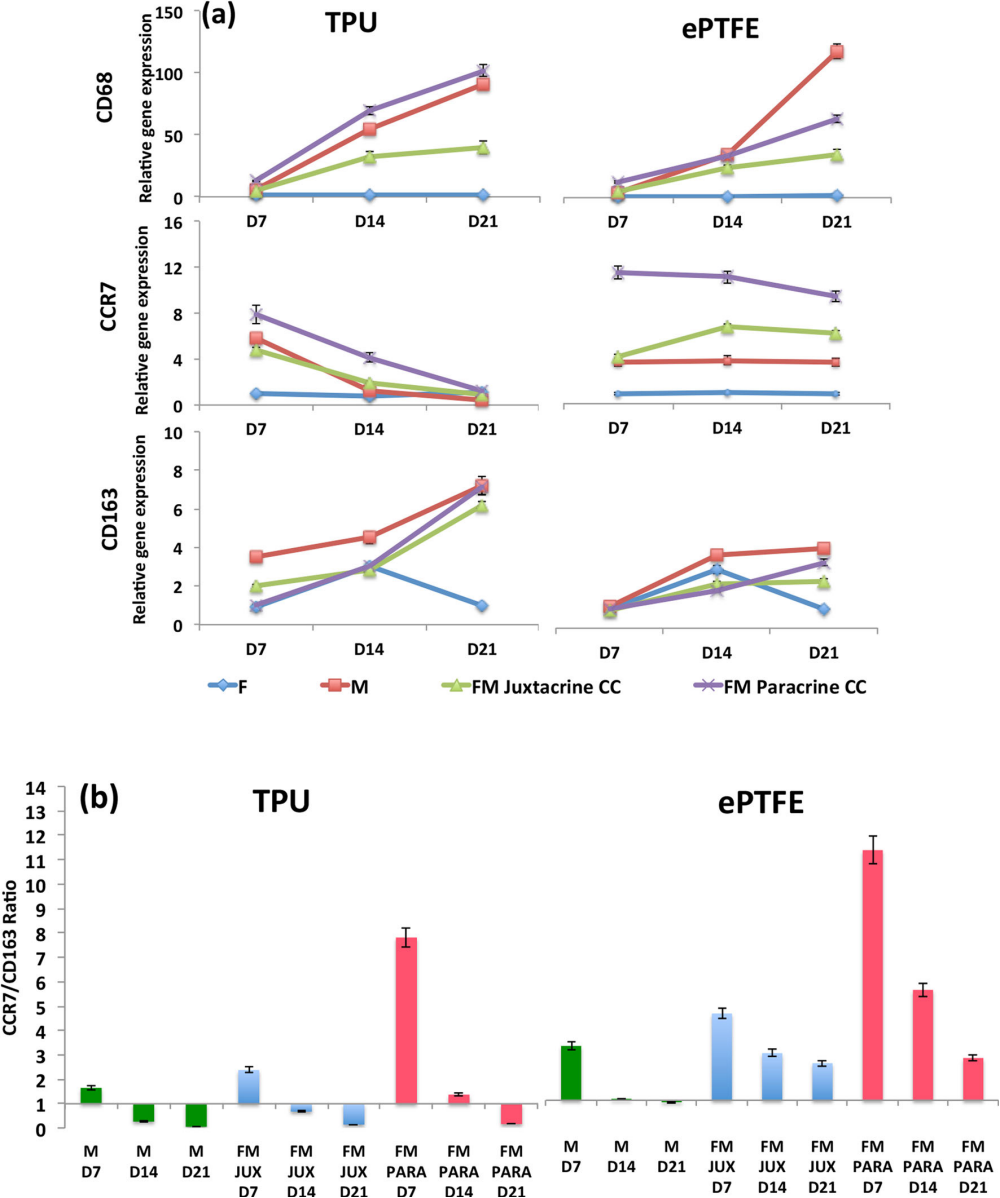
(a) CD68, CCR7 and CD163 macrophages gene expression studies in macrophage and fibroblast mono-cultures and fibroblast-macrophage juxtacrine (FM Juxtacrine CC) and paracrine co-culture (FM Paracrine CC) models, after 7, 14 and 21 days in TPU and ePTFE grafts. The expression levels of all marker genes were normalized to the expression levels of GAPDH. Data are expressed as mean ± SD (*n* = 3 per time-point per group, technical replicates: 3). (b) The ratios of CCR7/CD163 are illustrated for TPU and ePTFE grafts for macrophages mono-cultures (M) and fibroblast-macrophage juxtacrine (FM JUX) and paracrine co-culture (FM PARA) models at different time points.

Expression of pan macrophages (CD68), pro-inflammatory macrophages (CCR7) and anti-inflammatory macrophages (CD163) were apparent in TPU and ePTFE grafts after 7, 14 and 21 days (Fig. 4a). However, fibroblast mono-cultures revealed minor expressions of all these macrophage markers.

#### Comparison of FM Co-cultures vs. Macrophage Mono-cultures

In ePTFE grafts, CCR7 pro-inflammatory expression was significantly higher at all time points in both juxtacrine and paracrine co-cultures compared with macrophage mono-cultures. While, in TPU grafts, only FM paracrine co-cultures expressed significantly higher CCR7 genes (*p* < 0.05) compared with macrophage mono-cultures after 7 and 14 days. The expression of anti-inflammatory CD163 macrophages was not statistically different in macrophage mono-cultures compared with FM co-cultures.

#### Comparison of FM Paracrine vs. Juxtacrine Co-cultures

Expressions of pro-inflammatory macrophages (CCR7) and pan macrophages (CD68) in paracrine co-culture models were significantly higher than in juxtacrine models at all time points in both ePTFE and TPU grafts (except after 21 days, CCR7 in TPU). All these confirmed that in FM co-cultures especially in paracrine co-cultures, pro-inflammatory macrophages have been more activated compared to M mono-cultures.

#### Comparison of TPU vs. ePTFE Grafts

High level of pro-inflammatory (CCR7) was present in both grafts at day 7. This expression decreased significantly in TPU grafts (*p* < 0.001), whereas, it remained up-regulated in ePTFE grafts at all time points in macrophage mono-cultures and fibroblast-macrophage co-cultures. Furthermore, the level of anti-inflammatory CD163 in TPU grafts was significantly higher than in ePTFE grafts at all time points in M and FM culture models (*p* < 0.05).

The CCR7/CD163 ratios were further calculated using the correspondent gene expression levels for each graft type. TPU grafts initially had M1 pro-inflammatory responses, however after 14 days there was a clear switch from M1 pro-inflammatory (CCR7) to M2 anti-inflammatory (CD163) in all cultures, confirming a constructive response in TPU grafts (Fig. 4b). The initial pro-inflammatory response in TPU grafts in paracrine co-cultures was significantly higher than in juxtacrine co-cultures. In ePTFE grafts, FM co-culture models, showed predominant M1 pro-inflammatory responses however, a negligible transition from M1 toward M2 response was observed in M mono-cultures.

### *In-Vivo* Studies

#### Graft Retrieval and Macrophage Infiltration

**Figure 5:**
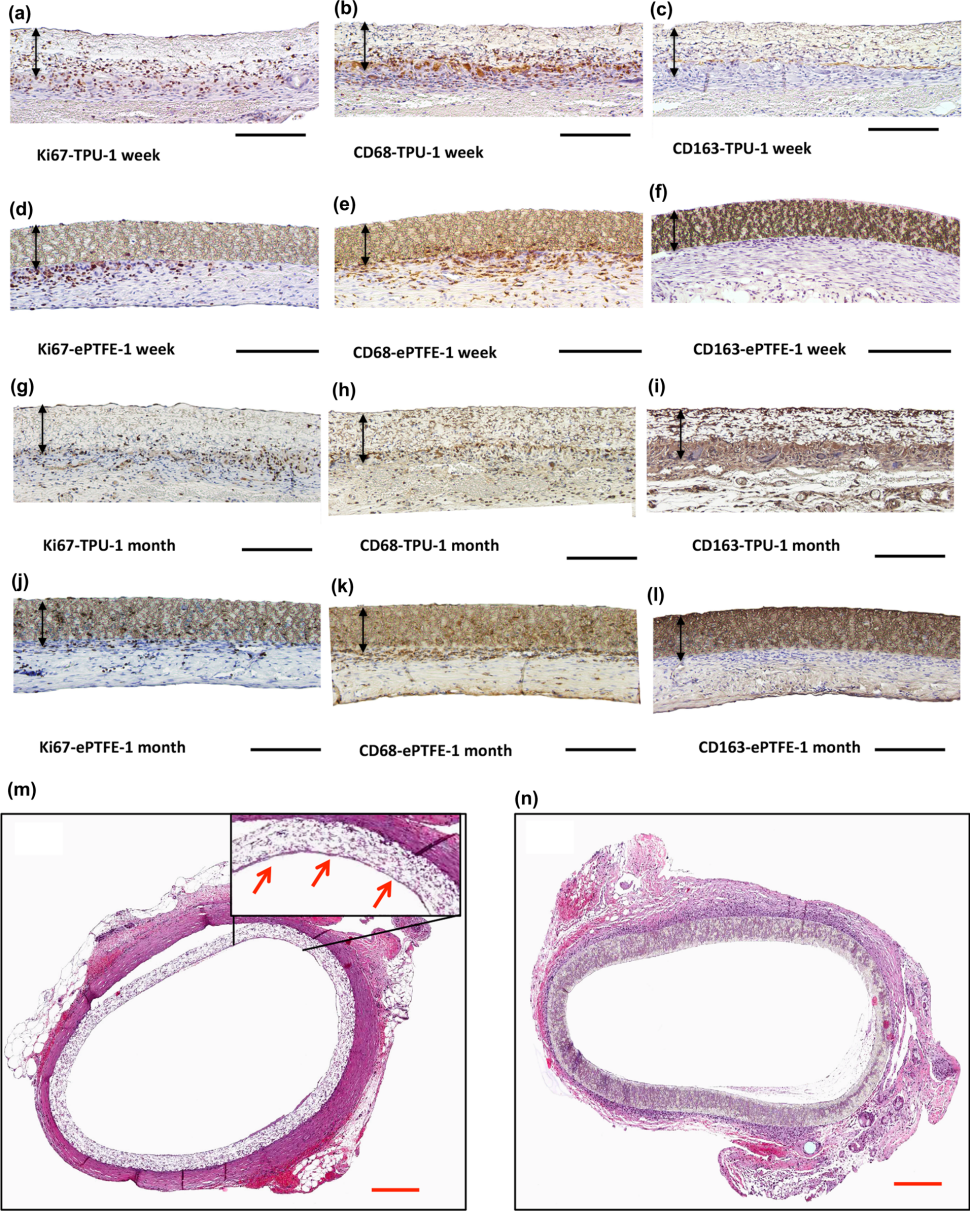
Immunohistochemical staining for (a, d, g, j) Ki67, (b, e, h, k) CD68, (c, f, i, l) CD163 for TPU and ePTFE grafts after 1 week and 1 month implantation. Black arrows indicate graft walls, black scale bar: 200 *µ*m. H&E stained of the cross-section of the (m) TPU and (n) ePTFE grafts after 1 month implantation, red arrows point out the luminal endothelial layer, red scale bar: 50 *μ*m.

After implantation and blood reperfusion, TPU graft wall was rapidly soaked with blood without signs of leakage. Upon retrieval, all of the prostheses (TPU, ePTFE) were patent. Gross microscopy showed adventitial tissue integration in TPU grafts (Supplementary Fig. 2). The porous structure of the TPU graft allows cell migration and proliferation, resulting in a highly cellular graft after 1 month implantation compared with ePTFE grafts. Both grafts had positive Ki67 and pan macrophages (CD68^+^) within the graft wall after 1 week and 1 month implantation. However, much of the positive cells in ePTFE grafts were only in the adjacent host tissue. The presence of M2 macrophage positive cells (CD163^+^), migrated inside the ePTFE grafts, was limited (Figs. 5a–5l). Prostheses showed no gross evidence of dilatation or aneurysmal formation. H&E staining revealed endothelial cells on the luminal surface of the mid-graft region of TPU grafts after 1 month. EPTFE grafts showed mononuclear cell infiltration at the tissue-implant interface (Fig. 5m, n). The inflammatory behavior of these grafts was further quantified *via* qPCR studies.

#### *In-Vivo* Biocompatibility Evaluation and Gene Expression Studies

**Figure 6:**
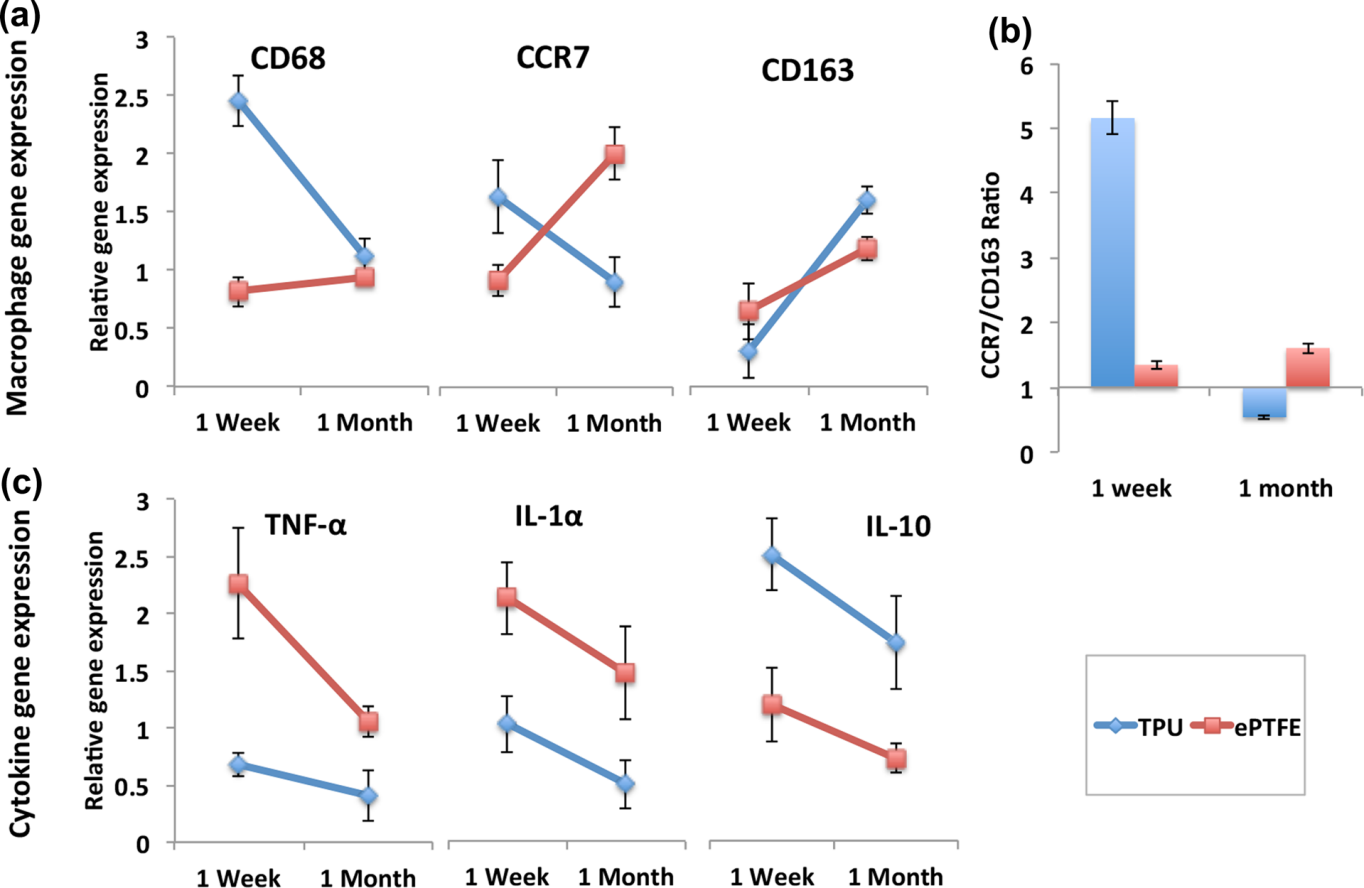
(a) CD68 and CD163 macrophages gene expression studies and (b) CCR7:CD163 ratio are illustrated for TPU and ePTFE grafts after 1 month and 1 week implantation. (c) Cytokines gene expression studies in TPU and ePTFE grafts after 1 week and 1 month implantation. The expression levels of all marker genes were normalized to the expression levels of GAPDH. The data are presented quantitatively as mean ± standard deviation (*n* = 4 per time-point per group, technical replicates: 3).

Gene expression of pro- and anti-inflammatory macrophages and cytokines were investigated for TPU and ePTFE grafts after 1 week and 1 month implantations. In TPU grafts, the level of anti-inflammatory CD163 up-regulated significantly (*p* < 0.001), while the level of pan macrophages (CD68) and pro-inflammatory macrophages (CCR7) down-regulated significantly (*p* < 0.05) (Fig. 6a). In ePTFE grafts, expression of CD68 and CD163 did not change significantly after 1 week and 1 month, whereas, the expression of CCR7 significantly up-regulated (*p* < 0.05). The calculated CCR7/CD163 ratios showed the predominant M1 pro-inflammatory response in ePTFE grafts. While, in TPU grafts, there was a clear switch from M1 pro-inflammatory to M2 anti-inflammatory response (Fig. 6b).

Cytokines release monitoring, revealed that only in TPU grafts, expressions of TNF-α and IL-1α pro-inflammatory cytokines were significantly lower than IL-10 anti-inflammatory cytokine after 1 month implantation. Comparing TPU vs. ePTFE grafts, expressions of pro-inflammatory TNF-α and IL-1α cytokines were significantly higher in ePTFE grafts, whereas level of IL-10 was significantly lower in these grafts after 1 month (Fig. 6c, (*p* < 0.05)).

#### Quantification and Phenotypic Characterisation of the Proliferated Cells

**Figure 7:**
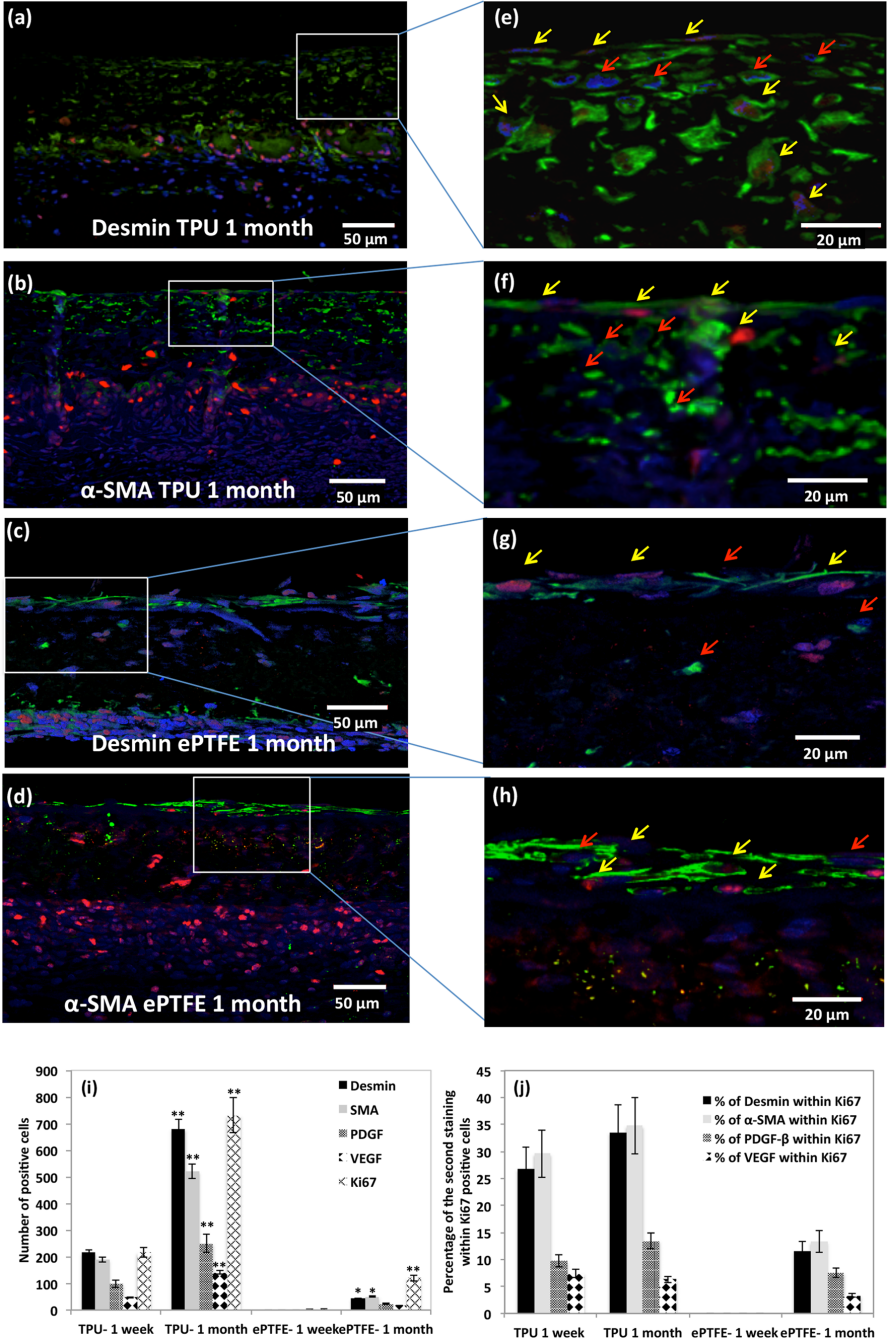
(a, b, c and d) Representative images of double immunofluorescence stained serial sections of TPU and ePTFE grafts after 1 month implantation showing host cell infiltration of Ki67^+^ cells (red), α-SMA^+^ and desmin^+^ cells (in green). Nuclei counterstained with DAPI (blue), scale bar: 50 *μ*m. (e,f,g and h) Magnified view of mid graft region; red arrows: ki67^−^ and desmin^+^/α-SMA^+^ cells, yellow arrows: co-expression of ki67-α-SMA or ki67-desmin of the infiltrated cells, scale bar: 20 *μ*m. (i) Quantification of the number of desmin^+^, α-SMA^+^, PDGF^+^, VEGF^+^, Ki67^+^ cells which migrated into the graft wall of TPU and ePTFE specimens (1 week, 1 month implantation, *n* = 4). Data are expressed as mean ± SD. *(*p* < 0.05) ** (*p* < 0.001) indicate statistical differences in cell populations between the two time points in each individual graft. (j) Percentage of desmin^+^, α-SMA^+^, PDGF^+^, VEGF^+^ cells within Ki67^+^ cells (*n* = 4).

Ki67^+^ cells mainly originated from adventitia in both grafts (Fig. 7). The population of the proliferated cells increased significantly from 1 week to 1 month only in TPU grafts (*p* < 0.001). Only TPU grafts showed α-SMA^+^, VEGF^+^, PDGF^+^, desmin^+^ cells after 1 week implantation. Desmin^+^ and α-SMA^+^ cells were evenly distributed within TPU grafts walls after 1 month. The population of these cells was significantly higher in TPU grafts compared with ePTFE grafts at all time points (*p* < 0.001). The luminal layer in TPU grafts composed of cells, mostly positive to VEGF after 1 month implantation (supplement Fig. 3). Population of VEGF^+^ and PDGF^+^ cells were significantly higher in TPU compared to ePTFE grafts at all time points. Co-expressions of Ki67^+^ cells, alongside with the other stainings (α-SMA, VEGF, PDGF, desmin), represented the type of the proliferated cells (Fig. 7). Co-expression of Ki67-desmin, Ki67-α-SMA, Ki67-PDGF and Ki67-VEGF were evident in the cells populating the TPU and ePTFE grafts after 1 month. The percentage of desmin^+^ and α-SMA^+^ cells within Ki67^+^ cell population was significantly higher than the percentage of Ki67-VEGF and Ki67-PDGF positive cells in TPU grafts after 1 week and 1 month of implantation.

## Discussion

Promoting cell infiltration and regulating inflammatory response by an implanted material are the two major factors for positive remodeling outcome and successful tissue regeneration of degradable materials. However, it is evident that capability of the implanted material to modulate pro-inflammatory response of macrophages is a pre-requisite for cell infiltration and cell growth.^5^ In this study, primary macrophages in mono- and co-cultured with fibroblasts (paracrine and juxtacrine co-culture models) were recruited for evaluation of the recently developed biodegradable electrospun grafts.

TPU grafts had superior support for fibroblast and macrophage cell attachment and proliferation, compared to ePTFE grafts in both mono- and co-cultured systems. This could be due to individual surface chemistry of the grafts and less hydrophobicity of TPU compared to ePTFE.^4^ Macrophages stimulated the proliferation of fibroblasts in co-cultures. It is known that activated macrophages induce fibrogenic cytokines such as IL1-α, TNF-α, PDGF and FGF, which stimulate fibroblast growth and proliferation.^8^,^24^ Cytokine release studies showed that early expression of the IL-1α and TNF-α was evident in both TPU and ePTFE grafts and this could enhance fibroblast proliferation in the co-culture.

The inflammatory phase of healing is associated with a significant up-regulation of the inflammatory cytokines, especially IL-6, TNF-α, IL-1α and IL-1β.^14^ These cytokines could be involved in different processes such as evoking migration/proliferation of fibroblasts, deposition/degradation of ECM and regulation of foreign body response.^23^ In constructive remodeling, the initial burst of inflammatory cytokines and M1 response is followed by a transitional phase toward M2 response, which is associated with significant down-regulation of pro-inflammatory cytokines. This phase stimulates the formation of scar tissue and inhibits chronic inflammation.^5^,^13^ In our study, initial expression of M1 macrophages (CCR7) and pro-inflammatory cytokines (TNF-α, IL-1α) was prominent in both macrophage mono-culture and in fibroblast-macrophage co-cultures in TPU and ePTFE grafts. Paracrine co-culture models showed higher pro-inflammatory response compared with juxtacrine co-culture models. After this initial inflammatory response, only TPU grafts modulated macrophage polarization toward M2 phenotype. The extent of this modulation was significantly higher in the co-culture models especially in paracrine co-culture models. It was shown previously that expression of IL-1α and TNF-α occurs considerably earlier than 72 h in macrophage-involved cultures.^23^ In our *in vitro* investigation, significant expression of pro-inflammatory cytokines (IL-1α, TNF-α) was evident after 2 h. This early phase was followed by significant down-regulation of TNF-α after 48 h. This shows the possible capability of fibroblasts and macrophages to modulate the inflammatory response to a foreign implant *via* expression of IL-10 anti-inflammatory cytokine. This expression has a key role in limiting the synthesis of pro-inflammatory cytokines such as TNF-α, IL-3 and IL-2.^7^

Following the adherence of macrophages to the implanted grafts in a rat model, the initial expression of M1 occurs in both TPU and ePTFE grafts. However, only TPU grafts ultimately promoted macrophage phenotypic transition towards M2 after 1 month implantation to support tissue repair and to inhibit chronic inflammation. Expression of inflammatory cytokines (TNF-α and IL-1α) was significantly higher than anti-inflammatory cytokines (IL-10) in ePTFE grafts. Continuous release of these inflammatory cytokines may lead to chronic inflammation and chronic wounding *in vivo*.^8^,^10^,^24^ Co-cultures had higher fidelity to *in vivo* situation compared to individual mono-cultures. Among the co-cultures, paracrine models better represented the *in vivo* initial pro-inflammatory response compared to juxtacrine models in TPU grafts. They also better represented the extent of the immunomodulatory effect of the biomaterial on macrophage polarization.

Remodeling of a biodegradable graft in terms of endothelialization and ECM production is highly governed by the migration and infiltration of host cells into the construct. Migration of endothelial cells (from the anastomosis or from transmural capillary ingrowth) and endothelial progenitor cells in bloodstream, play major roles in endothelialization and antithrombotic characteristics of a vascular graft. As previously seen, TPU grafts were fully endothelialized after 1 month implantation.^4^ Apart from cell infiltration, even distribution of cells throughout prosthesis is required to have uniform remolding and ECM deposition. It was shown that migration of the inflammatory cells and α-SMA^+^ cells is the major kind of migration in remolding of a vascular graft.^20^ Our previous study showed slight graft remodeling after 1 month implantation, however the extensive cellular repopulation of the grafts occurred after 12 months implantation in a rat model. The wall thickness did not change due to a precise balance between polymer degradation and tissue remodeling.^4^

TPU graft highly supported cell infiltration and proliferation compared to ePTFE grafts. Major cell infiltration in TPU grafts occurred *via* migration of macrophage, α-SMA^+^ and desmin^+^ cells. After 1 week implantation, CD68^+^ macrophages and Ki67^+^ proliferated cells were present in TPU grafts but mainly localized at adventitial layer. After 1 month, these cells were more evenly distributed throughout grafts at a lower density. Presence of CD163^+^ anti-inflammatory cells inside TPU grafts and very limited number of CD163^+^ cells in the adjacent tissue of the ePTFE grafts suggest the superior characteristic of TPU in recruiting healing macrophages compared to ePTFE grafts *in vivo*.

α-SMA^+^ and desmin^+^ cells are contractile filaments. They are considered as markers for developing vascular mural cells.^3^,^12^ Abundant number of α-SMA^+^ and desmin^+^ cells evenly distributed in the graft could indicate the regeneration of a contractile media in TPU grafts. Presence of PDGF and especially endothelium release of PDGF could be responsible in part for recruitment and proliferation of the vascular smooth muscle cells.^12^,^15^ Luminal layers of TPU and ePTFE grafts were comprised of VEGF^+^ cells after 1 month implantation. VEGF is considered as specific mitogen of the vascular endothelial cells, which promotes endothelial cell proliferation and migration. They also act as a major regulator of angiogenesis and vasculogenesis.^16^ VEGF^+^ cells were also present at the TPU host-tissue interface. It is well known that VEGF could be also produced by non-endothelial cells such as macrophages, platelets and cardiac myofibroblasts.^9^ Identifying the type of these VEGF^+^ non-endothelial cells remains to be studied. Quantification of the co-expression of Ki67-desmin, Ki67-α-SMA, Ki67-PDGF and Ki67-VEGF revealed that TPU grafts significantly supported the proliferation of α-SMA^+^ and desmin^+^ cells. These cells are primarily responsible for control of blood pressure and blood flow by responding to biomechanical stimuli.^17^,^21^

This study highlighted that macrophages in co-culture with fibroblasts in a paracrine model was a better representative of *in vivo* condition for assessing immunomodulatory potential of the TPU grafts. However, the absence of other inflammatory cells might confine the *in vitro* approach. Therefore, further assessing the biocompatibility of the graft and its influences on innate and acquired immunity such as T cells, B cells, natural killer cells and granulocytes should be considered in cell-biomaterial interaction studies.^19^ Furthermore, long-term functionality of the TPU graft should be assessed under low blood flow conditions in a large animal model. Comparing TPU graft with ePTFE graft, degradable TPU material showed desirable immunomodulatory effects on macrophages activation state and it promoted the macrophage polarization toward the M2 phenotype. It also highly supported cell infiltration, which could promote constructive remodeling.

## Conclusion

Morphology and structure of the TPU grafts supported attachment, viability and proliferation of the fibroblasts in mono and co-culture models. The extent of the inflammatory response was different in FM co-cultures compared to individual mono-cultures. Within the co-culture models, paracrine model better represented the initial pro-inflammatory response of the implanted TPU graft. Although, the co-culture models could not represent all the comprehensive aspects of the *in vivo* condition, it could provide a superior model to assess the *in vivo* behavior of the TPU graft prior to implantation. TPU grafts had superior characteristics in supporting cell infiltration and proliferation *in vivo*. A large number of α-SMA^+^ and desmin^+^ cells indicates the regeneration of a contractile media in TPU. TPU grafts also revealed an effective switch in macrophage polarization from M1 pro-inflammatory toward M2 anti-inflammatory *in vitro* and *in vivo*, which is a key component of constructive remodeling.

## Electronic Supplementary Material

Below is the link to the electronic supplementary material.

Supplementary material 1 (DOCX 2142 kb)
