# Supplementary material for: Biocompatibility Assessment of a New Biodegradable Vascular Graft viaIn Vitro Co-culture Approaches and In Vivo Model
Source: Ann Biomed Eng. 2016 Apr 7;44(11):3319–34. doi: 10.1007/s10439-016-1601-y (PMC5093217; doi:10.1007/s10439-016-1601-y)
Supplement: Supplementary file 1 — Supplementary material 1 (DOCX 2142 kb) [file 10439_2016_1601_MOESM1_ESM.docx]

**Supplementary Figures Captions:**

**Supplementary figure 1**. CV staining of macrophages and fibroblasts on (a) TPU & (b) ePTFE grafts after 7 and 21 days in F and M mono- and co-culture models.

**Supplementary figure 2.** Comparison of prostheses implanted into the infrarenal aorta. (a, c) TPU and ePTFE grafts immediately after reperfusion: TPU graft wall reddened with no evidence of blood leakage, ePTFE graft stayed white as it was prior the implantation. (b, d) Retrieval of prostheses after 1 month of implantation. Scale bar: 10 mm.

**Supplementary figure 3.** (a, b, c & d) PDGF^+^ and VEGF^+^ cells infiltration and organization after 1 month implantation as some representative images of double immunofluorescence staining on serial sections in TPU and ePTFE grafts, Ki67^+^ cells (red), PDGF^+^ and VEGF^+^ cells (in green) counterstained with DAPI (blue), scale bar: 50 μm. (e, f, g & h). Magnified view of mid graft region, Scale bar: 20 μm.
